# Anti-Inflammatory Effects of Vinpocetine in Atherosclerosis and Ischemic Stroke: A Review of the Literature

**DOI:** 10.3390/molecules20010335

**Published:** 2014-12-26

**Authors:** Linjie Zhang, Li Yang

**Affiliations:** Department of Neurology, Tianjin Neurological Institute, Tianjin Medical University General Hospital, 154, Anshan Road, Heping District, Tianjin 300052, China; E-Mail: linjie.zhang116@gmail.com

**Keywords:** vinpocetine, inflammatory response, ischemic stroke, atherosclerosis

## Abstract

Immune responses play an important role in the pathophysiology of atherosclerosis and ischemic stroke. Atherosclerosis is a common condition that increases the risk of stroke. Hyperlipidemia damages endothelial cells, thus initiating chemokine pathways and the release of inflammatory cytokines—this represents the first step in the inflammatory response to atherosclerosis. Blocking blood flow in the brain leads to ischemic stroke, and deprives neurons of oxygen and energy. Damaged neurons release danger-associated molecular patterns, which promote the activation of innate immune cells and the release of inflammatory cytokines. The nuclear factor κ-light-chain-enhancer of activated B cells κB (NF-κB) pathway plays a key role in the pathogenesis of atherosclerosis and ischemic stroke. Vinpocetine is believed to be a potent anti-inflammatory agent and has been used to treat cerebrovascular disorders. Vinpocetine improves neuronal plasticity and reduces the release of inflammatory cytokines and chemokines from endothelial cells, vascular smooth muscle cells, macrophages, and microglia, by inhibiting the inhibitor of the NF-κB pathway. This review clarifies the anti-inflammatory role of vinpocetine in atherosclerosis and ischemic stroke.

## 1. Introduction

Stroke is the second most common cause of death worldwide, and accounts for 6.7 million deaths (11.9% of total deaths) annually [1,2]. Ischemic stroke accounts for 80%–90% of all cases of strokes [3], and atherosclerosis is a common condition that increases the risk of stroke [3,4]. Atherosclerosis is a chronic inflammatory process affecting the large- and medium-size arteries, including those in the brain [5]. Large accumulation of lipids within artery walls stimulates a series of inflammatory responses—the stimulated endothelial cells attract T lymphocytes and monocytes, which transform into macrophages and ingest oxidized low-density lipoprotein (ox-LDL) to become foam cells. This complex structure, which includes subendothelial lipid accumulation, an increased number of extracellular matrix proteins, and diverse immune cell populations, is termed a plaque. In the microenvironment of inflammation, vascular smooth muscle cells (VSMCs) migrate and proliferate to form the cap of the plaque. Disruption of the cap attracts platelets, and coagulation proteins accumulate to form a thrombus. The development of such a lesion in the brain could lead to a thrombotic stroke and the traveling of an embolus to the brain could lead to an embolic stroke, which could cause ischemia in the area supplied by the affected artery [5,6,7]. The inflammatory pathophysiology of ischemic stroke has been studied for many years. Oxygen and glucose deprivation causes excitotoxicity, calcium overload, and oxidative stress, thus resulting in cell death in the infarct core [8]. Inflammation is involved in vasculitis, blood-brain barrier leakage, and further tissue damage. Oxidative stress causes the creation of inflammatory cells and the release of cytokines that together cause cellular damage in the infarct and peri-infarct area [9]. The damaged brain tissue releases inflammatory mediators that break down the blood-brain barrier and induce a robust inflammatory response in the lesion [10]. Activated microglia, the main immune cells in the central nervous system (CNS), release neurotransmitters and interact with neurons, thus contributing to post-ischemic inflammation. In addition, activated microglia and infiltrating macrophages contribute to tissue repair by removing dead cells [11]. Thus, the post-stroke inflammatory response is involved in tissue reconstruction and repair [12].

A key protein complex in the inflammatory response is the transcription factor nuclear factor κ-light-chain-enhancer of activated B cells κB (NF-κB), which is activated by a series of inflammatory molecules such as interleukin (IL)-6, IL-8 and tumor necrosis factor (TNF)-α. NF-κB initiates the expression of inflammatory cytokines and regulators of apoptosis. The NF-κB pathway includes NF-κB, the inhibitor κB (IκB), and IκB kinase (IKK). Ox-LDL in atherosclerosis is associated with NF-κB signaling in a dose-dependent manner, by upregulating the expression of proinflammatory genes [13]. In addition to the activation of endothelial cells and pathogenic proteins, the accumulation and proliferation of VSMCs are regulated via the NF-κB pathway in atherosclerosis. The role of the NF-κB pathway in ischemic stroke is complex and is still being investigated. Acute NF-κB activation was observed in an animal model of ischemic stroke (middle cerebral artery occlusion), and inhibition of NF-κB expression was associated with a reduced infarct size and decreased neurological deficits [14]. In the neurovascular unit, neuronal NF-κB inhibition reduced stroke size and apoptosis, while astroglial NF-κB inhibition had no effect [15]. Inhibition of the NF-κB pathway may provide a basis for the development of therapeutic strategies for the prevention and treatment of atherosclerosis and ischemic stroke.

**Figure 1:**
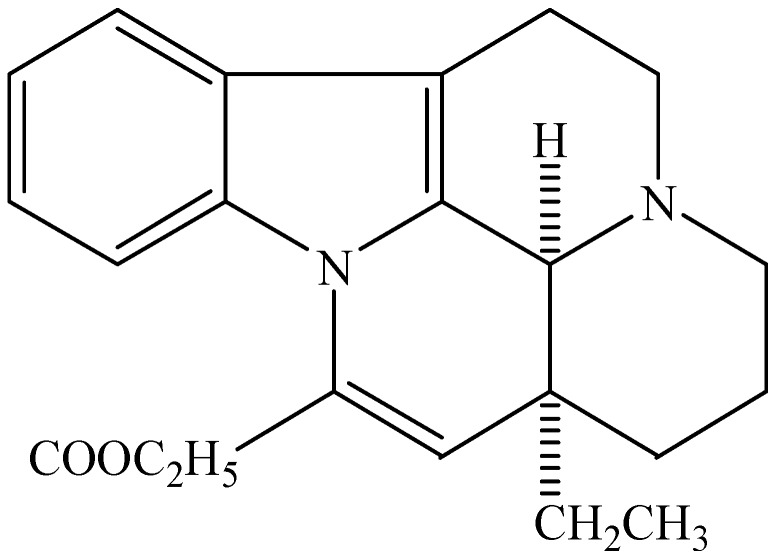
Chemical structure of vinpocetine.

Vinpocetine is an alkaloid extracted from the periwinkle plant, and is a derivative of the alkaloid vincamine. Its structure is shown in Figure 1 [16]. It has been used to enhance cerebral circulation and cognitive function for several years, and is currently being used in many countries as a dietary supplement to prevent cerebrovascular disorders and symptoms associated with aging [17,18]. Vinpocetine is an inhibitor of phosphodiesterase type 1 (PDE1), which can lead to increases in cAMP and cGMP, thus initiating plasticity-related gene expression [19]. Vinpocetine has a high affinity for the 18-kDa translocator protein (TSPO), which is a biomarker of activated microglia, and inhibits microglial proliferation through the NF-κB/activator protein-1 (AP-1) pathway. It also suppresses the release of inflammatory factors [20]. Vinpocetine suppressed the release of proinflammatory molecules by inhibiting the inhibitor of the IKK/NF-κB pathway after TNF-α stimulation [18], and also inhibits oligodendroglial precursor cell differentiation thus having a direct negative effect on remyelination [21]. This review will examine the evidence for the anti-inflammatory function of vinpocetine in atherosclerosis and ischemic stroke.

## 2. Anti-Inflammatory Effects of Vinpocetine in Atherosclerosis and Ischemic Stroke

### 2.1. Anti-Inflammatory Effects of Vinpocetine in Atherosclerosis

#### 2.1.1. Vinpocetine Inhibits the Progression of Atherosclerosis

**Figure 2:**
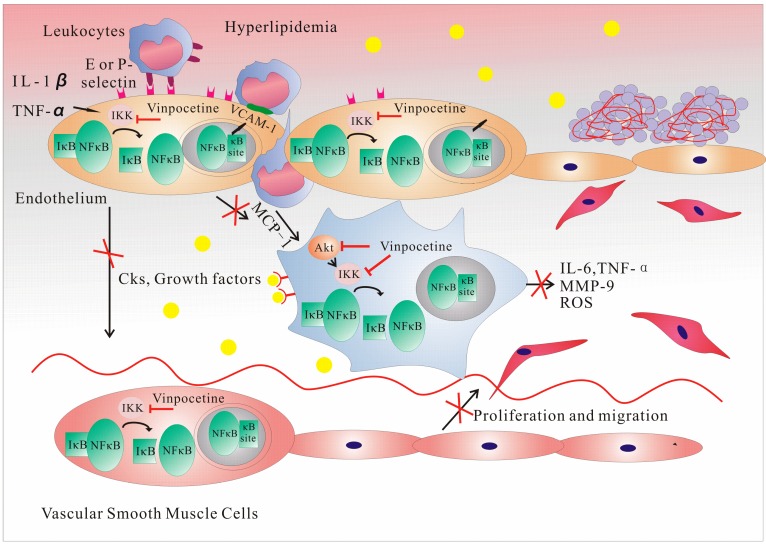
Vinpocetine inhibits the progression of atherosclerosis through the inhibition of IKK/NF-κB. Nuclear factor κ-light-chain-enhancer of activated B cells κB (NF-κB) is a protein complex that controls DNA transcription. It is a major transcription factor that regulates the genes responsible for both the innate and adaptive immune responses. In its inactive state, NF-κB is present in the cytoplasm, attached to the inhibitor of IκB kinase (IKK). When activated by inflammatory signals, the IKK complex phosphorylates and thus activates the inhibitor of κB (IκB). When IκB does not bind to specific DNA sequences (called response elements) to regulate translation, NF-κB is activated and translocates into the nucleus. This results in the triggering of adhesion molecules and cytokines, such as interleukin (IL)-1, IL-2, IL-6, and tumor necrosis factor-α, as well as the activation of chemotaxis. Vinpocetine inhibits the expression of vascular cell adhesion molecule-1 and the E- and P-selectins through the inhibition of NF-κB in endothelial cells, resulting in reduced blood leukocyte attraction and recruitment. By inhibiting monocyte chemoattractant protein-1 production in endothelial cells, vinpocetine affects transformation of monocytes into macrophages, thus further reducing the production of proinflammatory factors through IKK in macrophages. Vinpocetine reduces the expression of growth factors in endothelial cells, which then suppresses the migration and proliferation of vascular smooth muscle cells. This inhibition on VSMCs proliferation and migration occurs through the phosphorylation inhibition of platelet-derived growth factor-BB-induced extracellular signal-regulated protein kinases 1 and 2, and by inhibiting IKK-induced proinflammatory factor production.

Atherosclerosis has been known as an inflammatory disease for many years and can lead to ischemia of the heart, brain, or extremities, resulting in an infarction [7]. Pathogen-associated molecular patterns (PAMPs) evoke the inflammatory response to atherosclerotic damage, and mononuclear phagocytes are important to this response [22]. An increase in the expression of vascular cell adhesion molecule-1 (VCAM-1) and P-selectin occurs in endothelial cells in the presence of ox-LDL and inflammatory cytokines [23]; this increase, in combination with E-selectin—which is only found on endothelial cells and is stimulated by the expression of P-selectin [24]—contributes to blood leukocyte recruitment [25]. The oxidation of lipoprotein and proinflammatory cytokines, such as IL-1β and TNF-α, activates NF-κB, thus inducing VCAM-1 gene transcription [26]. Through regulation by NF-κB, the production of chemokines, such as the monocyte chemoattractant protein-1 (MCP-1) and IL-8, aids in the recruitment of monocytes to the endothelial cells. By targeting the NF-κB pathways, vinpocetine inhibits the transcription of adhesion molecules, selectins, and proinflammatory cytokines, and consequently inhibits monocyte adhesion in human umbilical vein endothelial cells (HUVECs) [27,28]. MCP-1 is pivotal in the transformation of monocytes into macrophages [6], and through NF-κB, vinpocetine can indirectly impact this process. Proinflammatory cytokines, such as IL-6, TNF-α, MCP-1, matrix metallopeptidase 9, and reactive oxygen species (ROS), released by macrophages that have phagocytosed ox-LDL, are also inhibited by vinpocetine through NF-κB [18,29]. The endothelial cells secreting growth factors, such as platelet-derived growth factor (PDGF), promote the abnormal migration and proliferation of VSMCs, which can be inhibited by vinpocetine [27,30]. Extracellular signal-regulated protein kinases 1 and 2 (ERK1/2) and Akt both regulate VSMC growth and migration through distinct signaling pathways [31]. Cai and colleagues found that vinpocetine specifically inhibited PDGF-BB-induced ERK1/2 phosphorylation, but not Akt phosphorylation, thus inhibiting PDGF-induced VSMC proliferation and migration. In addition, vinpocetine markedly inhibited the intracellular ROS generation induced by PDGF [30]. Vinpocetine enhances the collagen content and significantly increases fibrous cap thickness, thus stabilizing the atherosclerotic plaque [29]. Although PDE1 was initially thought to be the target for vinpocetine [32], IKK/NF-κB and ERK1/2 appear to be the pathways inhibited by vinpocetine, by reducing the inflammatory response in VSMCs. Vinpocetine relaxes cerebral VSMCs, thus enhancing cerebral blood flow [33]. The above research indicates that vinpocetine is able to inhibit many steps in the progression of atherosclerosis, which is important for the prevention of stroke (Figure 2).

#### 2.1.2. Vinpocetine and the Adaptive Immune Response in Atherosclerosis

The involvement of the adaptive immune response in atherosclerosis is demonstrated by the presence of antibodies against ox-LDL epitopes in the sera of patients with atherosclerosis [34]. The innate immune response involves the secretion of chemokines and cytokines, along with antigen presentation, and initiates the adaptive immune response, which is important to lesion progression. In this process, dendritic cells specifically initiate antigen-specific immunity and promote the activation and polarization of T cells [22]. Most of the T cells in atherosclerotic lesions bear the CD3 and CD4 markers and the T-cell antigen receptor αβ+. CD8+ cytotoxic T cells are also abundant in the plaques, which may indicate their role in inflammation [35]. The secretion of cytokines such as interferon-γ, IL-2, TNF-α, and TNF-β by lesional T cells causes the activation of macrophages and vascular cells. Of these, TNF-α can initiate the NF-κB signaling pathway. Tregs are another potential contributor, and represent 1%–5% of all T cells in the atherosclerotic lesions. Tregs have anti-inflammatory and immune regulation functions. A reduced number of Tregs in unstable plaques suggests their protective role in atherosclerosis [36]. Recently, B cells have been implicated in the pathogenesis of atherosclerosis. Oligoclonal B cells are found in lesions and undergo antigen driven proliferation [37]. The NF-κB pathway is important in both innate and adaptive immunity and controls T cell development [38]. Vinpocetine acts as an anti-inflammatory agent through the NF-κB pathway and affects many types of cells involved in the formation of atherosclerotic lesions; however, the effect of vinpocetine on the T and B cells in disease development requires further study.

### 2.2. Anti-Inflammatory Effects of Vinpocetine in Ischemic Stroke

#### 2.2.1. Vinpocetine Inhibits Early Inflammation in Ischemic Stroke by Inhibiting IKK/NF-κB

Once the atherosclerotic plaque ruptures, blood clotting will lead to rapid enlargement and, eventually, complete obstruction of the cerebral blood vessels, which will cause ischemia in the brain. The post-stroke inflammatory response has been studied for many years [39]. In 2008, a Cochrane review reported that there was insufficient evidence to evaluate the effect of vinpocetine on survival or a patient’s future dependency on others for care and activities of daily living in patients suffering an acute ischemic stroke [40]. A 2014 multicenter clinical study in China demonstrated that vinpocetine can reduce the level of disability during the early phase of stroke and can improve quality of life and cognitive ability after a stroke [41]. 

Obstruction of focal cerebral blood flow leads to oxygen and glucose depletion and neuronal death. Early ischemia and reperfusion (I/R) causes hypoxia, changes in shear stress, and the production of oxidative stress in endothelial cells that leads to microvascular occlusions [11]. Studies have shown that vinpocetine selectively affects cerebral blood flow without influencing systemic circulation [42] and improves the metabolism of oxygen and glucose in the brain, thus enhancing tissue tolerance of anoxia [43]. Vinpocetine acts on IKK, upstream of NF-κB, and inhibits TNF-α-induced NF-κB activation and the subsequent induction of proinflammatory mediators in VSMCs and endothelial cells [18].

**Figure 3:**
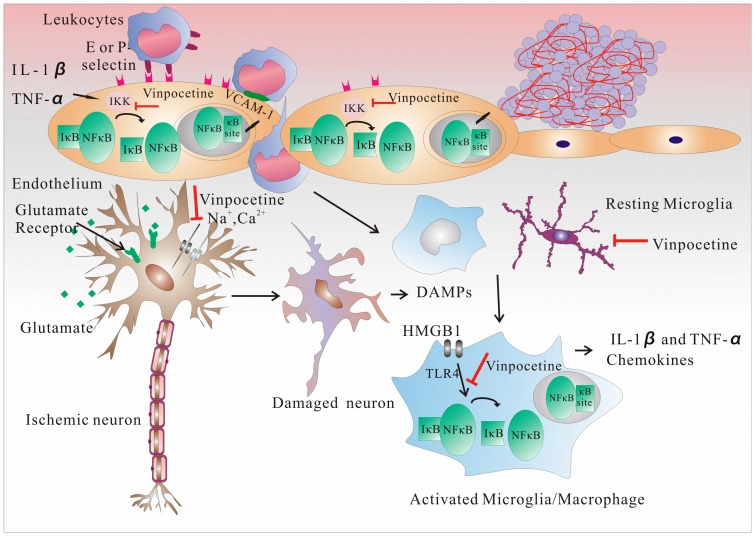
Vinpocetine inhibits the progression of stroke through the inhibition of IKK/NF-κB. Vinpocetine inhibits the expression of vascular cell adhesion molecule-1 and the E- and P-selectins through the inhibition of nuclear factor κ-light-chain-enhancer of activated B cells κB (NF-κB) in endothelial cells. This action protects the blood-brain barrier and reduces blood leukocyte attraction and recruitment. Vinpocetine protects neurons by inhibiting voltage-sensitive sodium channels and inhibiting cellular Ca^2+^ accumulation, which can lead to cell swelling and damage. Damaged neurons release danger-associated molecular patterns, which activate microglia and macrophages through Toll-like receptor 4 activation in the NF-κB pathway. Vinpocetine hinders this process by inhibiting inflammatory cytokine release and magnification. Although vinpocetine does not affect the activation of microglia, it inhibits the proliferation of microglia through NF-κB and the activator protein-1 transcription factors, which are major transcription factors that regulate the genes responsible for both the innate and adaptive immune responses and are important in the differentiation of T cells.

Ischemia-stimulated neurons release glutamate, thus activating glutamate receptors, which leads to a massive Na^+^ and Ca^2+^ influx. The increasing intracellular Na^+^ and Ca^2+^ concentrations in neurons are responsible for cell swelling and damage. High Ca^2+^ concentrations also activate Ca^2+^-dependent enzymes, thus resulting in oxidative stress. Vinpocetine selectively inhibits voltage-sensitive sodium (Na^+^) channels, thus inhibiting Ca^2+^ accumulation in the cells, and consequently inhibits neuronal damage. Vinpocetine also elicits an antioxidant effect in neurons [44]. TSPO is targeted during the preservation of mitochondrial functions, and may also be targeted when vinpocetine protects neurons [45]. However, the results indicate that vinpocetine does not exert its neuroprotective effects through this target [46]. Damaged neurons release danger-associated molecular patterns (DAMPs) that activate and infiltrate immune cells which secrete proinflammatory cytokines, thus contributing to neuron damage. DAMPs include ATP, nicotinamide adenine dinucleotide (NAD), heat shock protein (HSP), and high-mobility group box 1 protein (HMGB1). HMGB1 activates the Toll-like receptors (TLRs), especially TLR4, on microglia, perivascular macrophages, and brain endothelial cells [47]; blocking TLR4 activation and neutralizing monoclonal anti-HMGB1 antibodies results in smaller infarct sizes [48]. Vinpocetine inhibits the NF-κB pathway-mediated release of inflammatory cytokines, such as TNF-α, IL-1β, IL-12, and chemokines, which can, in turn, activate NF-κB to enhance the inflammatory reaction [49]. In the perivascular space, I/R causes perivascular macrophage activation, the release of proinflammatory mediators by macrophages (which contribute to the endothelial expression of adhesion molecules), and blood-brain barrier damage (which promotes the infiltration of leukocytes) [50]. Vinpocetine can inhibit this process [18]. At the same time, following I/R damage, neutrophils, macrophages, and microglia, including T cells, NK cells, and dendritic cells, infiltrate the lesion. T cells express and secrete chemokines, such as MCP-1, which attract cells to the damaged area [51]. After 4–6 h of ischemia, circulating leukocytes adhere to the vessel walls, leading to migration and accumulation in the ischemic brain lesion, which results in secondary injury. Neutrophils are highly associated with damage; inhibiting the adhesion molecules that facilitate neutrophil entry into the injured brain improves neurological outcomes [52]. This can be accomplished by vinpocetine’s inhibition of IKK/NF-κB in endothelial cells and macrophages [18]. The protective effect seen in lymphocyte-deficient mice, or caused by blocking postischemic trafficking of T cells into the ischemic brain, occurs 24–48 h after ischemia [53] (Figure 3).

#### 2.2.2. Vinpocetine Inhibits the Proliferation of Microglia by Inhibiting IKK/NF-κB

Microglia are the resident macrophages in the brain and spinal cord and act as the main active immune defense in the CNS, by modulating the neuroinflammatory processes. A key initial event in stroke is the rapid activation of resident immune cells, primarily microglia. Once activated, microglia produce inflammatory mediators and proteases, which exacerbate the ischemic damages in the brain, and at the same time exert a neuroprotective effect by phagocytosing dead cells and tissue debris and secreting neuroprotective factors, such as insulin-like growth factor [9,54]. Postischemic inflammation involves the activation and accumulation of microglia within the brain tissue, which lead to inflammatory injury [55]. Vinpocetine inhibits the proliferation of microglia through NF-κB/AP-1 and suppressed the release of inflammatory factors. Although microglia remove damaged cells, promote neurogenesis, induce the reestablishment of a functional neuronal environment through the restoration of the myelin sheath, and release neurotrophic factors and anti-inflammatory molecules, activated microglia also contribute to neuronal damage by producing NO, IL-1β, IL-6, and TNF-α. Vinpocetine inhibits the production of these molecules, and thus inhibits CNS inflammation [20]. When both lipopolysaccharide and oxygen-glucose deprivation-treated BV-2 microglia are exposed to vinpocetine-conditioned media, the death of primary cultured neurons declines markedly, thus demonstrating the neuroprotective effect of vinpocetine.

#### 2.2.3. Vinpocetine and the Adaptive Immune Response after Ischemic Stroke

Studies have found that the adaptive immune response plays an important role in post-ischemic stroke, and classic adaptive immunity reportedly contributes to ischemic brain injury. γδT cells release IL-17, which contributes to injury, whereas Treg cells have a protective role during the late stages of ischemic stroke; however, the role of Tregs in stroke is controversial [56]. NF-κB is a major transcription factor that regulates the genes responsible for both the innate and adaptive immune responses, and is important in the differentiation of T cells [38]. By influencing this transcription factor, vinpocetine can reduce the level of disability during the early phase of stroke, and can improve quality of life and cognitive ability after stroke [41]. More importantly, vinpocetine influences IKK/NF-κB in many cell types, thus reducing the release of inflammatory factors. Therefore, we hypothesize that vinpocetine also has an impact on post-stroke adaptive immunity. However, there is no direct evidence showing that vinpocetine affects the adaptive immune response, and this remains an area for future study.

Tissue reconstruction and repair occur following a stroke. These processes include the removal of dead cells, the development of an anti-inflammatory milieu, and the generation of prosurvival factors [11]. Microglia and infiltrating macrophages phagocytose dead cells and tissue debris after a stroke, and transforming growth factor-β and IL-10—which are promoted by phagocytosis—promote the resolution of inflammation and exert direct cytoprotective effects on the surviving cells. These steps suggest that vinpocetine may affect many steps in the post-stroke process. However, vinpocetine’s impact on the adaptive immune response and its mechanism of action in adaptive immunity require further study. 

## 3. Conclusions

Inflammation and immunity are involved in lesion formation in both atherosclerosis and ischemic stroke, and the NF-κB pathway plays an important role in their progression. Vinpocetine influences many steps in atherosclerosis and ischemic stroke by affecting endothelial cells, VSMCs, macrophages, and microglia, and by inhibiting the release of many inflammatory mediators by suppressing the IKK/NF-κB pathway. Thus, vinpocetine exerts a neuroprotective effect through anti-inflammatory mechanisms. The adaptive immune response has emerged as a contributor to the pathogenesis of atherosclerosis and ischemic stroke, and the activation and development of T and B cells in the adaptive immune response are influenced by NF-κB. However, the effect of vinpocetine on adaptive immunity and the adaptive immune response requires further study.

## Abbreviation

AP-1: activator protein-1
CNS: central nervous system
DAMPs: damaged neurons release danger-associated molecular patterns
ERK1/2: extracellular signal-regulated protein kinases 1 and 2
HUVECs: human umbilical vein endothelial cells
HSP: heat shock protein
HMGB1: high-mobility group box 1 protein
IL: interleukin
I/R: ischemia and reperfusion
TNF: tumor necrosis factor
IκB: inhibitor κB
IKK: IκB kinase
MCP-1: monocyte chemoattractant protein-1
NF-κB: nuclear factor κ-light-chain-enhancer of activated B cells κB
NAD: nicotinamide adenine dinucleotide
ox-LDL: oxidized low-density lipoprotein
PDE1: phosphodiesterase type 1
PAMPs: pathogen-associated molecular patterns
PDGF: platelet-derived growth factor
TLRs: Toll-like receptors
ROS: reactive oxygen species
TSPO: translocator protein
VCAM-1: vascular cell adhesion molecule-1
VSMCs: vascular smooth muscle cells

## References

[1] WHO The Top 10 Causes of Death. http://www.who.int/mediacentre/factsheets/.

[2] Lozano R., Naghavi M., Foreman K., Lim S., Shibuya K., Aboyans V., Abraham J., Adair T., Aggarwal R., Ahn S.Y. (2012). Global and regional mortality from 235 causes of death for 20 age groups in 1990 and 2010: A systematic analysis for the global burden of disease study 2010. Lancet.

[3] Go A.S., Mozaffarian D., Roger V.L., Benjamin E.J., Berry J.D., Borden W.B., Bravata D.M., Dai S., Ford E.S., Fox C.S. (2013). Heart disease and stroke statistics–2013 update: A report from the american heart association. Circulation.

[4] Adams H.P., Bendixen B.H., Kappelle L.J., Biller J., Love B.B., Gordon D.L., Marsh E.E. (1993). Classification of subtype of acute ischemic stroke. Definitions for use in a multicenter clinical trial. Toast. Trial of org 10172 in acute stroke treatment. Stroke.

[5] Ross R. (1999). Atherosclerosis–An inflammatory disease. N. Engl. J. Med..

[6] Libby P. (2002). Inflammation in atherosclerosis. Nature.

[7] Hansson G.K. (1999). Inflammation and immune response in atherosclerosis. Curr. Atheroscler. Rep..

[8] Candelario-Jalil E. (2009). Injury and repair mechanisms in ischemic stroke: Considerations for the development of novel neurotherapeutics. Curr. Opin. Investig. Drugs.

[9] Macrez R., Ali C., Toutirais O., le Mauff B., Defer G., Dirnagl U., Vivien D. (2011). Stroke and the immune system: From pathophysiology to new therapeutic strategies. Lancet Neurol..

[10] Endres M., Engelhardt B., Koistinaho J., Lindvall O., Meairs S., Mohr J.P., Planas A., Rothwell N., Schwaninger M., Schwab M.E. (2008). Improving outcome after stroke: Overcoming the translational roadblock. Cerebrovasc. Dis..

[11] Iadecola C., Anrather J. (2011). The immunology of stroke: From mechanisms to translation. Nat. Med..

[12] Murray K.N., Buggey H.F., Denes A., Allan S.M. (2013). Systemic immune activation shapes stroke outcome. Mol. Cell. Neurosci..

[13] Dabek J., Kulach A., Gasior Z. (2010). Nuclear factor κ-light-chain-enhancer of activated b cells (NF-κB): A new potential therapeutic target in atherosclerosis?. Pharmacol. Rep..

[14] Xu L., Zhan Y., Wang Y., Feuerstein G.Z., Wang X. (2002). Recombinant adenoviral expression of dominant negative IκBα protects brain from cerebral ischemic injury. Biochem. Biophys. Res. Commun..

[15] Zhang W., Potrovita I., Tarabin V., Herrmann O., Beer V., Weih F., Schneider A., Schwaninger M. (2005). Neuronal activation of NF-κB contributes to cell death in cerebral ischemia. J. Cereb. Blood Flow Metab..

[16] Ning M., Zhou Y., Chen G., Mei X. (2011). Preparation and *in vitro*/*in vivo* evaluation of vinpocetine elementary osmotic pump system. Adv. Pharmacol. Sci..

[17] (2002). Vinpocetine. Monograph. Altern. Med. Rev..

[18] Jeon K.I., Xu X., Aizawa T., Lim J.H., Jono H., Kwon D.S., Abe J., Berk B.C., Li J.D., Yan C. (2010). Vinpocetine inhibits NF-κB-dependent inflammation via an ikk-dependent but pde-independent mechanism. Proc. Natl. Acad. Sci. USA.

[19] Beavo J.A. (1995). Cyclic nucleotide phosphodiesterases: Functional implications of multiple isoforms. Physiol. Rev..

[20] Zhao Y.Y., Yu J.Z., Li Q.Y., Ma C.G., Lu C.Z., Xiao B.G. (2011). Tspo-specific ligand vinpocetine exerts a neuroprotective effect by suppressing microglial inflammation. Neuron Glia Biol..

[21] Torres K.J., Gottle P., Kremer D., Rivera J.F., Aguirre-Cruz L., Corona T., Hartung H.P., Kury P. (2012). Vinpocetine inhibits oligodendroglial precursor cell differentiation. Cell. Physiol. Biochem..

[22] Ammirati E., Moroni F., Magnoni M., Camici P.G. (2014). The role of T and B cells in human atherosclerosis and atherothrombosis. Clin. Exp. Immunol..

[23] Dong Z.M., Chapman S.M., Brown A.A., Frenette P.S., Hynes R.O., Wagner D.D. (1998). The combined role of p- and e-selectins in atherosclerosis. J. Clin. Investig..

[24] Leeuwenberg J.F., Smeets E.F., Neefjes J.J., Shaffer M.A., Cinek T., Jeunhomme T.M., Ahern T.J., Buurman W.A. (1992). E-selectin and intercellular adhesion molecule-1 are released by activated human endothelial cells *in vitro*. Immunology.

[25] Poole J.C., Florey H.W. (1958). Changes in the endothelium of the aorta and the behaviour of macrophages in experimental atheroma of rabbits. J. Pathol. Bacteriol..

[26] Collins T., Cybulsky M.I. (2001). NF-κB: Pivotal mediator or innocent bystander in atherogenesis?. J. Clin. Investig..

[27] Berdeaux A., Loueslati E., Gerard J.L., Pussard E., Giudicelli J.F. (1988). Evaluation of the natriuretic and beta-adrenoceptor-blocking effects of tienoxolol in normal volunteers. Fundam. Clin. Pharmacol..

[28] Park H.J., Zhang Y., Georgescu S.P., Johnson K.L., Kong D., Galper J.B. (2006). Human umbilical vein endothelial cells and human dermal microvascular endothelial cells offer new insights into the relationship between lipid metabolism and angiogenesis. Stem Cell Rev..

[29] Zhuang J., Peng W., Li H., Lu Y., Wang K., Fan F., Li S., Xu Y. (2013). Inhibitory effects of vinpocetine on the progression of atherosclerosis are mediated by AKT/NF-κB dependent mechanisms in apoe-/-mice. PLoS One.

[30] Cai Y., Knight W.E., Guo S., Li J.D., Knight P.A., Yan C. (2012). Vinpocetine suppresses pathological vascular remodeling by inhibiting vascular smooth muscle cell proliferation and migration. J. Pharmacol. Exp. Ther..

[31] Gerthoffer W.T. (2007). Mechanisms of vascular smooth muscle cell migration. Circ. Res..

[32] Bonoczk P., Gulyas B., Adam-Vizi V., Nemes A., Karpati E., Kiss B., Kapas M., Szantay C., Koncz I., Zelles T. (2000). Role of sodium channel inhibition in neuroprotection: Effect of vinpocetine. Brain Res. Bull..

[33] Szilagyi G., Nagy Z., Balkay L., Boros I., Emri M., Lehel S., Marian T., Molnar T., Szakall S., Tron L. (2005). Effects of vinpocetine on the redistribution of cerebral blood flow and glucose metabolism in chronic ischemic stroke patients: A pet study. J. Neurol. Sci..

[34] Goncalves I., Gronholdt M.L., Soderberg I., Ares M.P., Nordestgaard B.G., Bentzon J.F., Fredrikson G.N., Nilsson J. (2005). Humoral immune response against defined oxidized low-density lipoprotein antigens reflects structure and disease activity of carotid plaques. Arterioscler. Thromb. Vasc. Biol..

[35] Lichtman A.H., Binder C.J., Tsimikas S., Witztum J.L. (2013). Adaptive immunity in atherogenesis: New insights and therapeutic approaches. J. Clin. Investig..

[36] De Boer O.J., van der Wal A.C. (2010). FOXP3^+^ regulatory T cells in vulnerable atherosclerotic plaques. Int. J. Cardiol..

[37] Burioni R., Canducci F., Saita D., Perotti M., Mancini N., de Marco D., Clementi N., Chieffo A., Denaro M., Cianflone D. (2009). Antigen-driven evolution of b lymphocytes in coronary atherosclerotic plaques. J. Immunol..

[38] Gerondakis S., Fulford T.S., Messina N.L., Grumont R.J. (2014). NF-κB control of T cell development. Nat. Immunol..

[39] Magnus T., Wiendl H., Kleinschnitz C. (2012). Immune mechanisms of stroke. Curr. Opin. Neurol..

[40] Bereczki D., Fekete I. (2008). Vinpocetine for acute ischaemic stroke. Cochrane Database Syst. Rev..

[41] Liu N., Wei W.Z.W., Wei Y., Yin W. (2014). Efficacy and safety of vinpocetine in treatment of patients with acute cerebral infarction. Chin. J. Neuromed..

[42] Patyar S., Prakash A., Modi M., Medhi B. (2011). Role of vinpocetine in cerebrovascular diseases. Pharmacol. Rep..

[43] Karpati E., Szporny L. (1976). General and cerebral haemodynamic activity of ethyl apovincaminate. Arzneimittel-Forschung.

[44] Santos M.S., Duarte A.I., Moreira P.I., Oliveira C.R. (2000). Synaptosomal response to oxidative stress: Effect of vinpocetine. Free Radic. Res..

[45] Veenman L., Gavish M. (2006). The peripheral-type benzodiazepine receptor and the cardiovascular system. Implications for drug development. Pharmacol. Ther..

[46] Tarnok K., Kiss E., Luiten P.G., Nyakas C., Tihanyi K., Schlett K., Eisel U.L. (2008). Effects of vinpocetine on mitochondrial function and neuroprotection in primary cortical neurons. Neurochem. Int..

[47] Marsh B.J., Williams-Karnesky R.L., Stenzel-Poore M.P. (2009). Toll-like receptor signaling in endogenous neuroprotection and stroke. Neuroscience.

[48] Tang S.C., Arumugam T.V., Xu X., Cheng A., Mughal M.R., Jo D.G., Lathia J.D., Siler D.A., Chigurupati S., Ouyang X. (2007). Pivotal role for neuronal toll-like receptors in ischemic brain injury and functional deficits. Proc. Natl. Acad. Sci. USA.

[49] Zotti T., Uva A., Ferravante A., Vessichelli M., Scudiero I., Ceccarelli M., Vito P., Stilo R. (2011). TRAF7 protein promotes Lys-29-linked polyubiquitination of IκB kinase (IKKγ)/NF-κB essential modulator (NEMO) and p65/Rela protein and represses NF-κB activation. J. Biol. Chem..

[50] Konsman J.P., Drukarch B., van Dam A.M. (2007). (Peri)vascular production and action of pro-inflammatory cytokines in brain pathology. Clin. Sci..

[51] Worthmann H., Tryc A.B., Goldbecker A., Ma Y.T., Tountopoulou A., Hahn A., Dengler R., Lichtinghagen R., Weissenborn K. (2010). The temporal profile of inflammatory markers and mediators in blood after acute ischemic stroke differs depending on stroke outcome. Cerebrovasc. Dis..

[52] Zheng Z., Yenari M.A. (2004). Post-ischemic inflammation: Molecular mechanisms and therapeutic implications. Neurol. Res..

[53] Kleinschnitz C., Schwab N., Kraft P., Hagedorn I., Dreykluft A., Schwarz T., Austinat M., Nieswandt B., Wiendl H., Stoll G. (2010). Early detrimental t-cell effects in experimental cerebral ischemia are neither related to adaptive immunity nor thrombus formation. Blood.

[54] De Oliveira A.C., Candelario-Jalil E., Langbein J., Wendeburg L., Bhatia H.S., Schlachetzki J.C., Biber K., Fiebich B.L. (2012). Pharmacological inhibition of akt and downstream pathways modulates the expression of COX-2 and mPGES-1 in activated microglia. J. Neuroinflamm..

[55] Wang Q., Tang X.N., Yenari M.A. (2007). The inflammatory response in stroke. J. Neuroimmunol..

[56] Kleinschnitz C., Kraft P., Dreykluft A., Hagedorn I., Gobel K., Schuhmann M.K., Langhauser F., Helluy X., Schwarz T., Bittner S. (2013). Regulatory T cells are strong promoters of acute ischemic stroke in mice by inducing dysfunction of the cerebral microvasculature. Blood.

